# Protein-Losing Enteropathy in Primary Lymphangiectasia

**DOI:** 10.5334/jbsr.2136

**Published:** 2020-06-29

**Authors:** Bernard Crutzen, Pierre-Antoine Poncelet

**Affiliations:** 1 Cliniques Universitaires Saint-Luc, BE

**Keywords:** Lymphangectasia, waldmann, lymphangioma, enteropathy, hyponatremia, protein

## Abstract

**Teaching Point:** Intestinal lymphangectasia should be evoked in the rare context of protein-losing enteropathy with low-attenuation thickening of the bowel wall.

## Case Report

**Figure 1:**
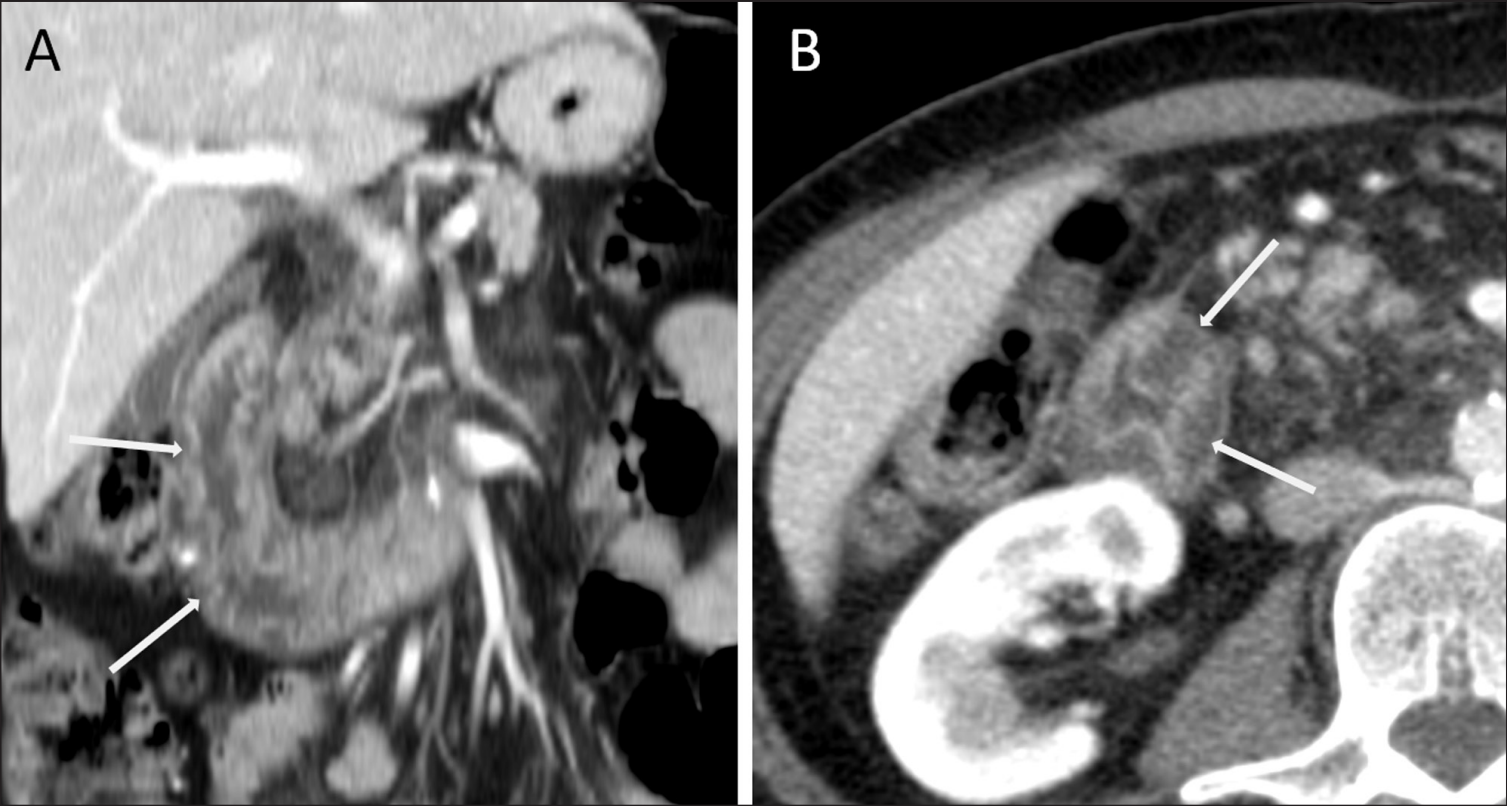


A 54-year-old female was hospitalized for severe hyponatremia and electrolytic troubles. She had increasing fatigue, loss of appetite and lower limbs edema. Abdominal contrast-enhanced computed tomography (CT) revealed circumferential parietal thickening of the proximal duodenum, with low attenuation of the submucosal layer (Figure 1A and B, arrows).

There was no fat infiltration nor vessel hypertrophy suggestive of inflammatory bowel disease. Endoscopic exploration showed mucosal ulcerative lesion and the biopsy revealed focal lymphangiectasias in the submucosal layer. Clinical presentation associated with biological and histological findings suggested the diagnosis of protein-losing enteropathy (PLE). Human serum albumin scintigraphy (Tc99m) confirmed the diagnosis and showed loss of tracker in the duodenum.

**Figure 2:**
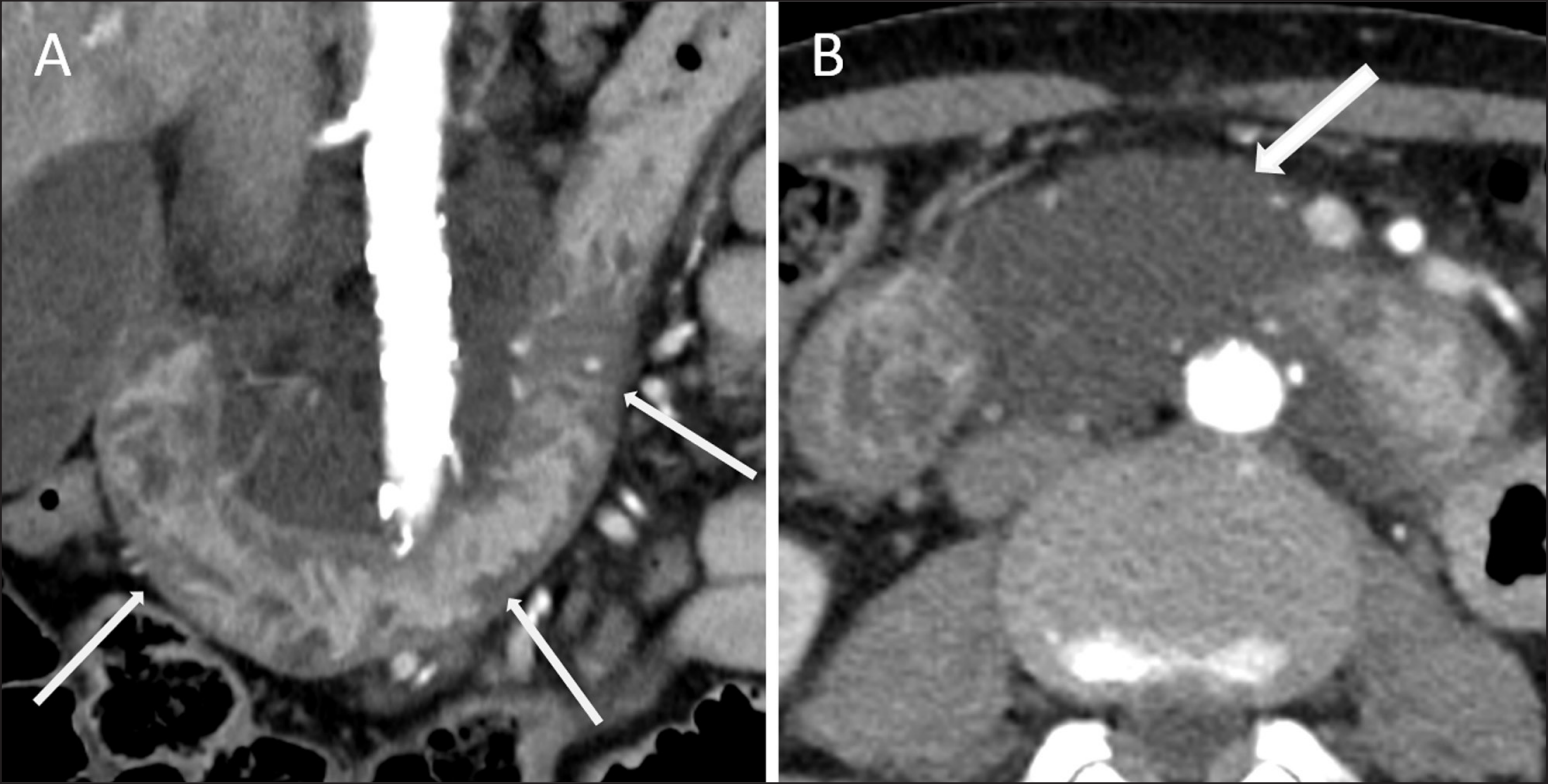


Eight years later, a repeat CT showed extension of the low-attenuation thickening to the whole duodenum (Figure 2A, arrows), and a 10-centimeter mesenteric cystic mass extending to the liver hilum without tissue infiltration (Figure 2B, arrow).

The radiological features and its localization highly suggested the diagnosis of cystic lymphangioma. Final diagnosis was primary intestinal lymphangiectasia, also known as Waldmann’s disease.

## Discussion

Waldmann’s disease or primary intestinal lymphangiectasia is usually diagnosed in children, but few cases have been reported in adults. It is characterized by lymphatic dilatation and results in excessive loss of proteins into the intestinal lumen leading to lower limbs edemas, diarrhea, and ascites. Findings of lymphangiectasia on CT include bowel wall thickening with hypoattenuating submucosa, corresponding to dilated lymphatic channels [1]. Development of lymphangioma is uncommon. Differential diagnosis includes inflammatory bowel disease, graft versus host disease, Whipple disease, and infections. Absence of sign of acute inflammatory disease as fat infiltration and hyperemia can be helpful to guide the diagnosis.

Lymphangiectasia is a difficult diagnosis on imaging but should be evoked in presence of other lymphatic abnormalities, especially in the context of protein-losing enteropathy.
